# Rare Primate *Rhinopithecus bieti* Can Sustain the Resilience of Montane Forests

**DOI:** 10.3390/ani15203021

**Published:** 2025-10-17

**Authors:** Na Li, Hao-Han Wang, Yan-Peng Li, Cyril C. Grueter, Lu-Jiao Dai, Hui-Ming Xu, Zhi-Pang Huang, Wen Xiao

**Affiliations:** 1 Institute of Eastern-Himalaya Biodiversity Research, Dali University, Dali 671003, China; lin@eastern-himalaya.cn (N.L.);; 2 Yunling Black-and-White Snub-Nosed Monkey Observation and Research Station of Yunnan Province, Dali 671003, China; 3 Collaborative Innovation Center for Biodiversity and Conservation in the Three Parallel Rivers Region of China, Dali 671003, China; 4 The Provincial Innovation Team of Biodiversity Conservation and Utility of the Three Parallel Rivers Region from Dali University, Dali 671003, China; 5 Center for Interdisciplinary Sciences, Dali University, Dali 671003, China; 6 Research Center of Natural History and Culture, Qujing Normal University, Qujing 655011, China; 7 School of Human Sciences, The University of Western Australia, Perth, WA 6009, Australia; 8 International Centre of Biodiversity and Primates Conservation, Dali 671003, China; 9 Bureau of Nuozhadu Provincial Nature Reserve, Lancang, Pu’er 665600, China; 10 Bureau of Tianchi National Nature Reserve, Yunlong, Dali 672700, China

**Keywords:** rare species, ecological function, canopy structure, under-canopy climate, surrogate species, intermediate disturbance

## Abstract

**Simple Summary:**

Understanding how rare animals contribute to ecosystem health is crucial for conservation. This study explored how the endangered black-and-white snub-nosed monkey helps maintain forests in southwestern China. These monkeys break branches and create gaps in tree canopies, which scientists think might strengthen forests by altering light and temperature patterns. Comparisons between currently used monkey habitats and those abandoned decades ago revealed that active habitats exhibited higher tree gap prevalence (38%) compared to abandoned sites (29~33%), which supported greater plant biodiversity. For example, plant species richness in current habitats is more than double that in areas abandoned for 40 years without monkey presence. Even 20 years after monkeys disappear, their past disturbances leave lasting benefits like higher plant diversity. This shows the monkeys play a vital role in keeping forests healthy and diverse over time. Protecting such rare species is therefore key to sustaining ecosystems, as their long-term effects help forests adapt to challenges like climate change. These findings stress the importance of safeguarding not just individual animals but also their unique ecological impacts for healthier environments.

**Abstract:**

This study addresses a critical challenge in global conservation: understanding how rare species contribute to ecosystem structure and resilience. The ecological role of the endangered black-and-white snub-nosed monkey in China’s temperate mountain forests was examined, with the hypothesis that its tree-shaking behavior alters forest structure and microclimates to enhance ecosystem health. To assess long-term impacts, current monkey-inhabited forests were compared with historical sites abandoned over decades, by analyzing tree gaps, forest structure, and environmental conditions. Monkeys’ canopy-disturbing actions were also directly observed. Findings revealed monkey activity created more canopy gaps (38.3% in current habitats vs. 29.9~33.5% in abandoned sites) and altered microclimate conditions, which boosted plant diversity and optimized the community’s vertical and age structures. Current forests supported nearly twice as many tree species, 2.5 times as many shrub species, and threefold more herb species than areas abandoned for 40 years. Even 20 years after monkeys disappeared, abandoned sites retained higher diversity and gaps, showing lasting ecological benefits. These results confirm the monkey’s vital role as a resilience promoter, demonstrating how rare species can shape healthier ecosystems. This highlights the need to prioritize protecting such species, as their survival not only preserves biodiversity but also sustains ecosystem functions crucial for human well-being.

## 1. Introduction

The role of rare species in ecosystems has been identified as one of the top 100 fundamental questions in ecology [1]. Whether rare species play a key role in ecosystems has long been debated. Many researchers ascribe crucial ecological functions to rare species in ecosystems [2]. To improve conservation efficiency, rare animals are often used as surrogate species, which are charismatic to the public, and as keystones, which exert disproportionately large effects on the ecological community. Others suggest that the function of rare species in an ecosystem is limited; e.g., Jordán emphasizes that rare species are less important in food webs on account of their rarity [3]. Thus, clarifying the ecological function of rare species, especially rare animals, is necessary for effective biodiversity conservation and natural ecosystem management. However, empirical evidence supporting the functional roles of rare species—particularly large mammals in biodiversity-rich mountain ecosystems—remains insufficiently documented. This is mainly due to the difficulty of conducting explicit tests of ecological function [4], which entails long-term field experiments [5] and species removal experiments [6]. Our current knowledge of the ecological function of rare species is mainly based on models using a trophic perspective [2,7,8]. Therefore, there is an urgent need to conduct targeted empirical studies on rare species.

The black-and-white snub-nosed monkey *Rhinopithecus bieti* is a member of the primate subfamily Colobinae and is listed as Endangered by the IUCN [9], threatened by human activity and population isolation induced by habitat fragmentation. According to our research team’s assessment, as of 2021, this monkey species has a total population of about 3500 individuals (unpublished data from Institute of Eastern-Himalaya Biodiversity Research). This species is endemic to China with a narrow distribution in the Yunling Mountains in the Mountains of Southwest China biodiversity hotspot [10]. The population size and status of this monkey are quite similar to those of Milne-Edward’s Sifaka *Propithecus edwardsi*, which is endemic to the eastern coastal rainforest of Madagascar and distributed in a narrow area and severely fragmented [11]. These species’ similar conditions suggest shared conservation challenges. As frugivorous arboreal primates, they may play critical roles in forest ecosystems. Understanding their ecological function could inform restoration strategies for both species and ecosystems. The black-and-white snub-nosed monkey has a relatively low birth rate; i.e., females begin to reproduce around 4–5 years old and give birth every second year thereafter, with 6–7-month gestation period [12], which limit the species’ ability to rapidly increase its population. The monkey has been designated as a flagship species to support regional biodiversity conservation due to its visually distinctive morphological traits, including striking ocular pigmentation, an upright fur pattern on the crown, and pronounced pinkish-red lip coloration. Though the monkey has low abundance and limited geographic range with specific habitat in a global context, they are the most dominant mammal in terms of biomass in a regional scale. The monkeys are the largest arboreal primates, with adult males weighing up to 30 kg. They occupy the highest elevational range of any nonhuman primate (2600–4600 m) and are associated predominantly with temperate conifer forests and mixed deciduous broadleaf and conifer forests [13,14]. This species is also one of the few mammals known to eat lichens all year round. The monkeys promote seed dispersal [15], and they rotate over large areas of their home range.

Forest canopy gaps play a critical role in facilitating ecologically sustainable forest management by inducing changes in key environmental factors such as light intensity and soil humidity. These alterations promote biodiversity preservation, enhance nutrient cycling efficiency, maintain the structural complexity of forest ecosystems, and support the regeneration of tree species, collectively reinforcing the adaptive capacity of forest ecosystems [16,17]. Forest managers must consider the role of natural disturbance in forest canopy dynamics. Through field observations, we documented that monkeys spend the majority of their time in the canopy, where they frequently break branches during movement and feeding. While such behaviors may contribute to canopy gaps, the ecological implications of these actions remain speculative. Therefore, it was proposed that the monkeys may act as agents of structural disturbance in temperate coniferous forests.

**Figure 1:**
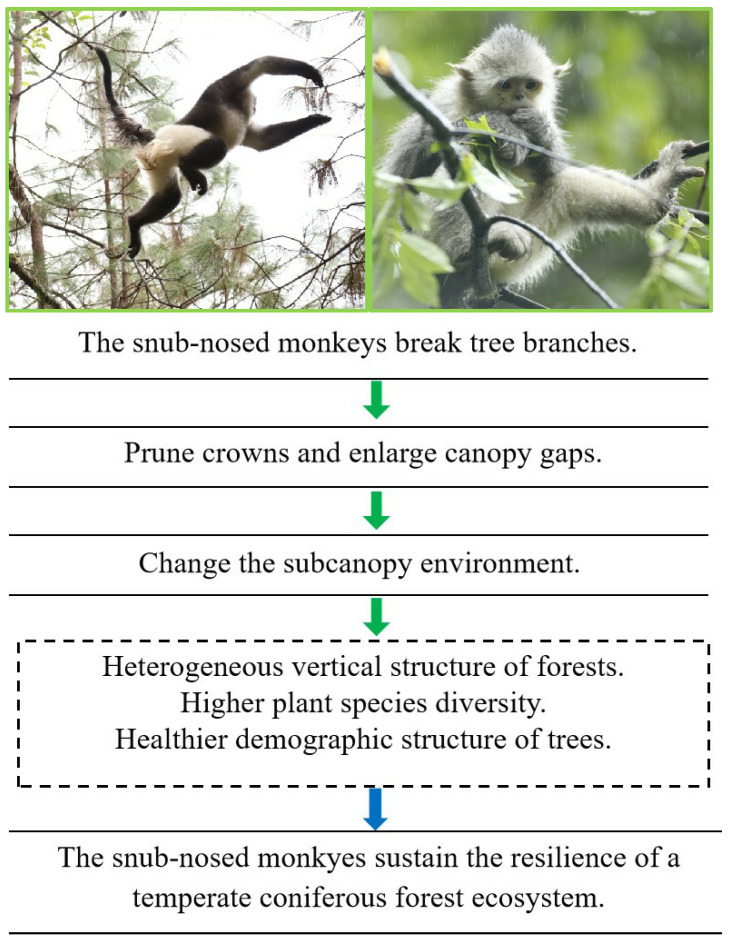
Hypotheses relating to the ecological role of the black-and-white snub-nosed monkey (*Rhinopithecus bieti*) tested in this study. The monkeys increase the size of canopy gaps by breaking branches during moving and feeding. Larger canopy gaps improve the water, solar and temperature conditions of the subcanopy, thus optimizing the forest structure. Collectively, the snub-nosed monkeys act as engineers by inducing disturbance to the canopy and thereby sustaining the resilience of the temperate coniferous forest ecosystem. Green arrows indicate ecological processes and blue arrow indicates conclusion from the ecological processes.

The monkey is thus hypothesized to play a pivotal ecological role in the temperate coniferous forest ecosystem for the following reasons: By breaking branches through feeding and moving, the monkey increases canopy gaps, which changes the sub-canopy environment and enhances structural heterogeneity and species diversity, i.e., resulting in more heterogeneous vertical stratification; higher species diversity across trees, shrubs, and herbs; and a healthier tree demographic structure. Figure 1 systematically summarizes the above-mentioned ecosystem function hypotheses regarding *R. bieti* and the data content that needs to be collected for hypothesis testing. This hypothesis was tested by quantitatively measuring the monkey’s branch-breaking behavior, and comparing the canopy structure, sub-canopy environment and forest community structure between current habitats and historical habitats where the monkey has successively disappeared over the last 40 years.

## 2. Materials and Methods

### 2.1. Study Area

The study area (25°50′ N–26°25′ N; 99°15′ E) is located at the southern extent of the Three Parallel Rivers Region, Yunnan Province, China (Figure S1). The Three Parallel Rivers Region is one of the richest regions in terms of biodiversity in the world. It also lies at the intersection of three global biodiversity hotspots: the Himalayas, the Indo-Myanmar region and the mountains of Southwest China [18]. The north–south range of the study area is 50 km, and the east–west range is 3 km. The study area encompasses temperate coniferous forests, which are underlain by Ferrisols, Luvisols, Spodosols, Cambisols, Entisols and Gleysols soils. The study area is characterized by a tree vegetation layer comprising mainly *Tsuga dumosa*, *Rhododendron* spp. and *Abies georgei*, and a shrub vegetation layer comprising mainly *Rhododendron faberi*, *Rhododendron mucronatum*, and *Gamblea ciliata var. evodiifolia*. Mean annual rainfall in the area is 1000–1800 mm, with a rainy season (June-October) and a dry season (November to May).

The study area marks the southernmost locality record for the black-and-white snub-nosed monkey, where the population has dwindled dramatically [19]. The study area contains the species’ current habitat, i.e., Lasha Shan (H-0), but also historical habitats, i.e., Jiayan (where local extinction occurred around 10 years ago; hereafter denoted by ‘H-10’), Yuanbao Shan (H-20), Tianzi Shan (H-30), and Tianchi and Xi Shan (H-40). Based on our interview-based surveys conducted in 2019, the monkey groups in these historical habitats vanished at different times: 2008 at Jiayan, 1997 at Yuanbao Shan, 1989 at Tianzi Shan, and 1980 at Tianchi and Xi Shan (Table S1). The surviving population in Lasha Shan consisted of about 100 individuals in 11 one-male multi-female units and two all-male units. In 2010, the one-male multi-female units consisted of 27 adult females, and 16 of them gave birth [20].

### 2.2. Observations of Branch-Breaking Behavior

To test our hypotheses, we first quantified the snub-nosed monkeys’ branch-breaking behavior using focal observations and field measurements at Lasha Shan in November 2018 (autumn), January 2019 (winter) and April 2019 (spring). Ten effective full-day follows were conducted in each season, with a total observation time of 11 h per day, from 07:40 to 18:40. While following the monkeys, the focal sampling and one-zero sampling method [21] was used to collect data on branch-breaking behavior by the monkeys. Based on observational experience, a 5 min interval focal sampling ensures capturing a sufficient number of events during periods of high behavioral occurrence frequency while avoiding the omission of sudden behavioral events that could be missed due to excessively prolonged intervals. Adult male or female individuals were selected alternately for 5 min focal observation sessions. For a continuous observation period of 5 min, we conducted continuous behavioral recording of the individual being observed. We recorded each behavior type, as well as the start and end times. We calculated the frequency of occurrence of each behavior on a daily basis. Young ones were not selected because they rarely break branches while feeding, and these individuals are relatively small and seldom break branches during jumping. When branches were broken as a result of utilization by the monkeys, we recorded the behavior and sex of the individual responsible for the damage as well as the diameter, length and number of broken branches.

### 2.3. Measuring the Structural Properties of Broken Branches

Whether the structural properties of branches broken by monkeys were different from those caused by other factors such as wind and small animals were tested. We have tracked the ~100 individual monkey population at Lasha Shan since 2008 and established the dimensions of their home range. In spring 2019, twenty 20 × 20 m sampling quadrats were established: ten within the monkey’s current habitat and ten within a 200 m buffer zone adjacent to the habitat boundary. Within each quadrat, fallen branches were first manually removed to standardize baseline conditions. Ten days later, all branches ≥ 1 cm in diameter were systematically collected, counted, and measured for diameter and length.

### 2.4. Measuring Forest Structure, Under-Canopy Environment and Broken Branches in Current and Historical Habitats

Forest structural parameters and branch characteristics were assessed at the five sites in the 65 quadrats (Figures S1 and S2), including the current habitat (H-0) where the monkeys have always lived, and four historical habitats where the monkeys vanished approximately 10 (H-10), 20 (H-20), 30 (H-30) and 40 years (H-40) ago. From October 2018 to March 2019, sampling quadrats (20 × 20 m) were set up in H-0, H-10, H-20, H-30 and H-40. In each of the five habitats, quadrats were set up in five elevation zones with an interval of 100 m from 3000 to 3400 m a.s.l. Due to topographical restrictions, quadrats in H-40 were established at the following elevations: 3000 to 3100 m at Tianchi and 3200 to 3400 m at Xi Shan. Mountain slopes were divided into four directions: north (0 ± 45 degrees), east (90 ± 45 degrees), south (180 ± 45 degrees) and west (270 ± 45 degrees). Southern slopes are heavily influenced by human activities and largely populated by Yunnan pine (*Pinus yunnanensis*) forest, which is generally avoided by the monkeys. Thus within each elevational zone, quadrats were systematically positioned on three cardinal slopes—north, east, and west. To control for the effect of human activity and natural disasters, sampling quadrats were set up in areas where felled timbers and stumps were free of cuts and saw marks and where there was no trace of debris flow, landslides and fire. Fifteen quadrats were established in H-0, H-10, H-20 and H-30, respectively (Figure S2). In H-40, only 13 quadrats were established because there is no western slope at 3100 m and no eastern slope at 3200 m (Figure S2). Due to the lack of data from western slopes at 3100 m and eastern slopes at 3200 m in H-40, all records from quadrats on western slopes at 3100 m and eastern slopes at 3200 m of the other four habitats were also excluded to standardize the sampling effort and make the data more intuitively comparable (as shown in Tables S2 and S3); in other words, data from only 65 quadrats were analyzed (5 habitats × 13 quadrats). The tree quadrats were 20 × 20 m. In addition, 5 × 5 m quadrats for shrubs and 1 × 1 m quadrats for herbs were established in the corners and center of each tree quadrat, respectively. The quadrats sampled in H-0 account for 0.043% of the entire home range of the resident monkey population. Monthly visits to all quadrats were conducted for patrol management and to gather meteorological data. Additionally, the assessment of the plant structure within each quadrat was performed once, during the spring of 2019.

The following variables among the five sites were quantified and compared (Table S2): (i) length and diameter of broken branches; (ii) crown diameter and canopy gap size; (iii) solar radiation, air temperature and air moisture under the canopy; (iv) vertical stratification of the plant community, including height of trees, shrubs and herbs; (v) species diversity of the plant community, including species richness and abundance of trees, shrubs and herbs; (vi) demographic structure of trees, including diameter at breast height, age structure, proportion of hollow trees, abundance of saplings and proportion of saplings without their mother trees nearby. Diameter and length of broken branches on the ground with a diameter greater than 1 cm were measured. Through sustained observational studies in this region, branches broken by monkeys were found to typically exceeded 1 cm in diameter, whereas those damaged by wind or smaller fauna (e.g., rodents) were consistently thinner than 1 cm. Trees with a height of less than 20 cm were considered as shrubs, trees with a height > 20 cm and a stem diameter at breast height (DBH, 1.3 m above the ground) < 1 cm were viewed as young saplings. Young saplings without adult trees of the same tree species in the quadrat were defined as motherless saplings. Hollowness is a manifestation of deteriorating health. Hollowness of trees is affected by many factors, such as age of trees and human disturbance. Since there was only slight human disturbance in our quadrats, the hollowness of trees was largely caused by age. Species name, height, crown diameter and DBH of trees with DBH > 10 cm were recorded in each quadrat. To measure the age structure of trees, the core of a tree was drilled out at a position of 1.5 m above the ground with a tree growth cone (HagLof, Sweden) with an inner diameter of 1.5 cm and a sampling length of 700 mm. The size of canopy gaps were measured by Fisheye hemisphere image analysis [22]. A Sony A6000 camera (Sony China Co., Ltd., Beijing, China) and a Samyang 12 mm Fisheye lens (Samyang Optical Industries Co., Ltd., Seoul, Republic of Korea) were used to take three photos horizontally from the center of each quadrat at a position of 1.5 m above the ground. The Gap Light Analyzer software version 2.0 was used to analyze the size of forest canopy gap in the quadrats. Measurements from the three photos were averaged to obtain a value for the size of a canopy gap in one quadrat. This methodological procedure is described in more detail in the Supporting Information (Table S4).

Lastly, the effects of canopy gaps on solar radiation, air temperature and air moisture under the canopy were examined, along with the impacts of these factors on the vertical structure, species diversity and demographic structure of the forest. From April to October 2019, solar radiation, air temperature and air moisture were measured in quadrats described in Figure S2 in H-0, H-10, H-20, H-30 and H-40. A photometer (McCo Technology Co., Ltd., Beijing, China), a thermometer (Jian Da Renke Electronic Technology Co., Ltd., Jinan, China) and a hygrometer (Jian Da Renke Electronic Technology Co., Ltd., Jinan, China) were installed in the center of the quadrat. Solar radiation, heat (air temperature) and water (air moisture) conditions were recorded every 30 min throughout the day from April to October 2019, a period that is important for the growth of plants. Based on the pre-experiment, a 30 min interval effectively captures changes in light intensity while balancing data recording density with storage capacity. For each of the five sites, the average value of solar radiation of days were calculated at 9:30 am (when it reaches its maximum value). Averages for air temperature and moisture condition were also calculated (Table S2). Mantel test [23] was used to analyze four environmental correlates of the plant community structure. An Environment × Site matrix and a Biological variable × Site matrix were built. Environmental factors were size of canopy gaps, solar radiation, air temperature, and air moisture; the biological variables were abundance, height, species richness of herbs, shrubs and trees, and abundance of all saplings and motherless saplings. The values are shown in Table S2. Pairs of environmental factors were compared with Spearman’s correlation tests. All analyses were performed in the R statistics programming environment [24] version 4.1.0 with the package “stats”.

### 2.5. Statistical Analysis

Considering the sample sizes of the data and whether they conform to a normal distribution, we conducted statistical tests on various variables between different habitats using the following methods (Table S4).

Welch two sample *t*-test for variables with a large sample size (n > 30) and with a normal distribution: Length of broken branches, Diameter of broken branches, Crown diameters, Canopy gaps, Solar radiation, Height of trees, Height of shrubs, Height of grass.

Paired Wilcoxon signed rank test for variables with a small sample size (n < 30), discontinuous data, or non-normal distribution: Species richness of trees, Species richness of shrubs, Species richness of grass, Density of trees, Density of shrubs, Density of grass, Diameter at breast height of trees, Proportion of hollow trees, Density of young saplings, Proportion of motherless young saplings.

## 3. Results

### 3.1. Canopy Pruning Behavior

A total of 6814 behavioral records were collected, such as moving, feeding, grooming, resting, fighting, and playing, of which 370 constituted instances of branch-breaking: 219 of these instances were caused by adult males, 149 by adult females, and 2 by juveniles. An adult monkey breaks an average of 12.3 branches a day based on daily observation session (52% caused by moving and 43% by feeding, 5% were associated with play and aggression), with an average length of 101.71 cm (SD = 79.46, n = 370) and a diameter of 2.04 cm (SD = 1.04, n = 370).

**Figure 2:**
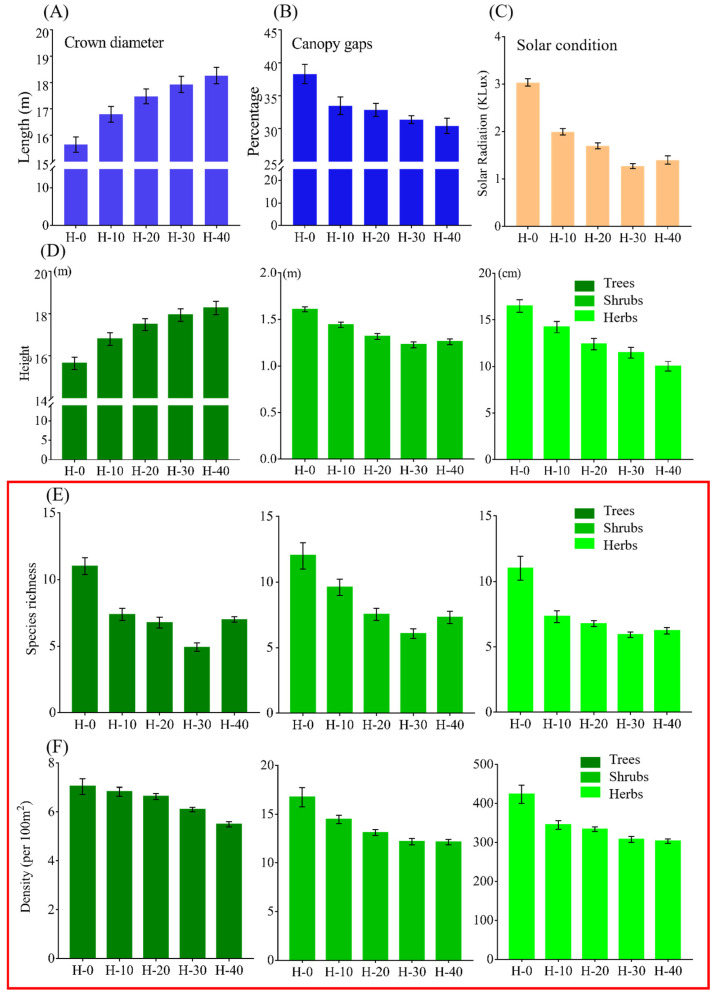
Black-and-white snub-nosed monkeys optimize the ecosystem community structure by widening canopy gaps (**A**,**B**), increasing solar radiation below the canopy (**C**), diversifying the vertical structure of forests (**D**), increasing the species diversity of plant communities (**E**,**F**, red box). H-0 is the currently used habitat by the monkeys; H-10, H-20, H-30 and H-40 are the four historical habitats from which the monkeys vanished 10, 20, 30 and 40 years ago, respectively. (**A**) Crown diameter in the current habitat was significantly smaller than in historical habitats. (**B**) The size of canopy gaps was positively correlated with crown diameter and was significantly wider in areas where the monkeys disappeared less recently. (**C**) Solar radiation below the canopy increased with increasing gap sizes. (**D**) The height of trees in the current habitat was shorter than in historical habitats and decreased significantly with increasing time since the extirpation of the local monkey population. The height of shrubs and grasses in the current habitat was longer than in historical habitats. (**E**) A greater diversity of trees, shrubs and grasses was found in current habitats. (**F**) The density of trees, shrubs and grasses showed a similar decreasing trend with increasing time since the monkeys’ extirpation. Error bars indicate ± SEM. All statistical details are shown in Table S4.

We collected 139 broken branches in Lasha Shan, 59 in H-0 and 80 near H-0. The average diameter of branches in H-0 was significantly larger than that of branches near H-0 (4.10 ± 2.03 cm vs. 1.80 cm ± 1.17; Wilcoxon rank sum test, W = 4121.5, *p* < 0.001). The average length of broken branches in H-0 was also significantly larger than that of branches near H-0 (157.59 ± 85.62 cm vs. 58.48 ± 19.37 cm; Wilcoxon rank sum test, W = 3993.5, *p* < 0.001). The number of broken branches with a diameter ≥ 2 cm accounted for 83% of the total broken branches in H-0, but only 21% in areas around H-0. We also found that the diameter and the length of broken branches in the current habitat were significantly greater than in historical habitats (Table S2; and refer to Table S4 for statistical details). These findings show that broken branches caused by factors other than monkey activity are thinner and shorter than those caused by monkey activity, and broken branches with a diameter ≥ 2 cm were mainly the result of monkey-induced damage. Because the removal of larger branches through breakage creates larger canopy gaps than the removal of smaller ones, and the removal of larger branches was mainly attributable to *R. bieti*, the monkey group had a greater effect on canopy architecture than other variables. Evidently, crown diameter in the monkeys’ current habitat was significantly smaller than in historical habitats (Tables S2 and S4), and crown diameter decreased significantly with canopy gap (Spearman correlation analysis, R^2^ = 0.962, *p* = 0.0032). That is, crown diameter increased significantly with increasing time since the local extirpation of the monkey population (Figure 2A; Table S2), while canopy gaps decreased correspondingly (38.30 ± 2.18%, 33.48 ± 2.35%, 32.85 ± 1.69%, 31.39 ± 0.67%, 29.87 ± 1.63%, respectively; *t*-test, *p* < 0.0001; Figure 2B; Table S2).

### 3.2. Sub-Canopy Environment

**Figure 3:**
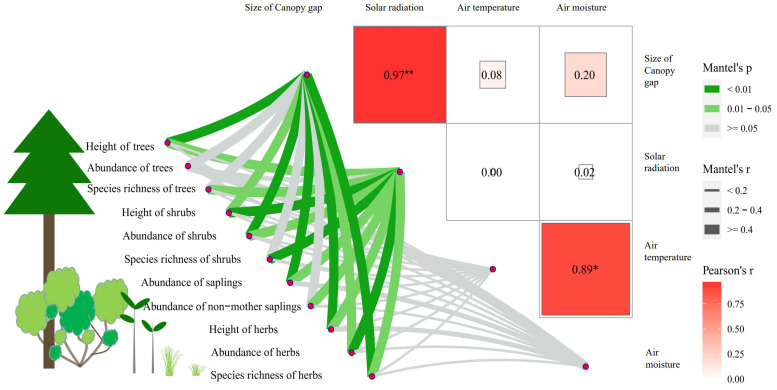
The effect of canopy gaps on solar radiation, air temperature and air moisture of the subcanopy and the effect of these factors on the abundance, height and species richness of herbs, shrubs and trees and the abundance of saplings and motherless saplings. The size of canopy gaps had a strong and significantly linear effect on solar radiation below the canopy (Spearman correlation analysis, R^2^ = 0.94, *p* < 0.0001), but not on air temperature and air moisture. The abundance, height and species richness of herbs and shrubs were strongly and significantly affected by the size of canopy gaps and solar radiation. The abundance of saplings and motherless saplings and the height and species richness of trees were also affected by canopy gaps and solar radiation. “*” indicates *p* < 0.05, and “**” indicates *p* < 0.01.

There was a strong positive correlation between the size of canopy gaps and solar radiation below the canopy (Figure 3). Air temperature and air moisture below the canopy are positively correlated, and these two variables both showed a tendency to rise first and then fall with increasing canopy gaps (Table S2; Figure 2C and Figure 3). This indicates that changes in temperature and humidity with gaps are more complex as they are influenced by a combination of light and airflow, as small gaps increase light and warmth without greatly increasing wind exposure/evaporation, while very large gaps lead to greater wind speed and vapor pressure deficit, reducing moisture.

### 3.3. Changes in Forest Structure and Vertical Stratification

**Figure 4:**
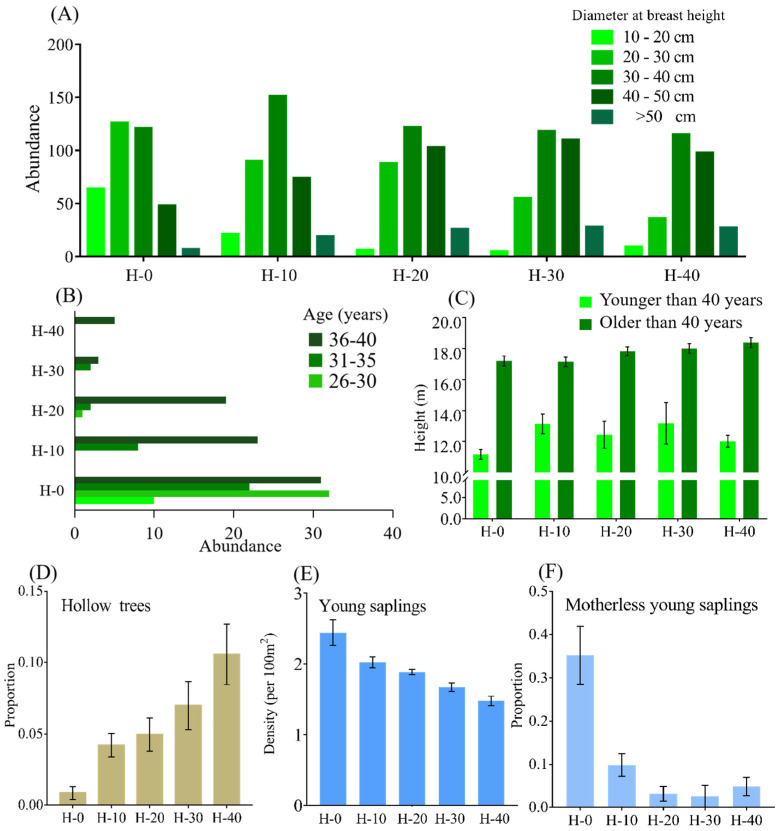
Comparing the demographic structure of trees between current and historical habitats. H-0 is the currently used habitat by the monkeys; H-10, H-20, H-30 and H-40 are the four historical habitats from which the monkeys vanished 10, 20, 30 and 40 years ago, respectively. (**A**) Analyses of the diameter at breast height and (**B**) the age structure of trees show that the number and proportion of younger trees was smaller in areas where snub-nosed monkeys vanished a long time ago. The scarcity of trees under the age of 40 in historical habitats could be due to the fact that (**C**) they are shorter than older trees and narrower canopy gaps may increase the mortality of younger trees. In addition to the demography of trees, (**D**) the proportion of hollow trees increased while (**E**) the density of young saplings and (**F**) the proportion of motherless saplings decreased with increasing time since the extirpation of the monkeys. Error bars indicate ±SEM. All statistical details are shown in Table S4.

Dominant plant species were consistent in terms of species composition in all habitats (Table S3). The height of trees decreased with increasing size of canopy gaps, while shrubs and herbs showed the reverse trend (Figure 2D; Table S2), indicating a more heterogeneous vertical structure in H-0. Both species richness and abundance of stems of trees, shrubs and herbs increased with the size of canopy gaps (Figure 2E,F). Hence, a wider canopy facilitated an increase in the height, species richness and abundance of shrubs and grasses. The diameter at breast height of trees (Figure 4A) and the height heterogeneity of trees (Figure S3) had a similar structure across the five sites, which shows that the number and proportion of younger trees were smaller in areas where snub-nosed monkeys vanished a long time ago (Figure 4B). The scarcity of trees under the age of 40 in historical habitats could be because they are shorter than older trees (Figure 4C) and narrower canopy gaps may increase the mortality of younger trees [16]. The proportion of hollow trees, an indicator of a tree’s health status, increased with decreasing size of canopy gaps (Figure 4D). The abundance of young saplings (Figure 4E) and the proportion of saplings without their mother trees nearby (Figure 4F) also increased with increasing canopy gaps, which suggests that a wider canopy promotes seedling survival and colonization of opportunistic colonizers [25,26]. In contrast to shrubs and grasses, canopy gaps had a delayed effect on tree density; the density of trees and young saplings at H-10 and H-20 was not significantly lower than at H-0, but that at H-30 and H-40 was. In addition, the monkeys themselves also promote seed dispersal through feeding and moving in this area as proven before [15], resulting in a higher number of young motherless saplings in the current habitat. The height, abundance, and species diversity of shrubs and herbs were strongly and significantly affected by canopy gaps and solar radiation, and the abundance of all saplings and motherless saplings (Figure 3). The height and species richness of trees were also significantly and strongly affected by canopy gaps and solar radiation (Figure 3). Thus, wider canopy gaps visibly improved the heterogeneity of the subcanopy structure and the diversity of trees. These positive effects of ecological engineering decreased 20 years after the monkeys vanished from the ecosystem. This is shown by the fact that the density of trees and young saplings at H-30 and H-40 (but not H-10 and H-20) was significantly lower than in the current habitat (Figure 2F and Figure 4E). For a summary of the above data, see Table S2; for additional statistical details, see Table S4.

## 4. Discussion

The role of particular species in the functioning of ecosystems has been identified as a question of fundamental importance for conservation practice and policy [27]. Moreover, Sutherland et al. emphasized that the functioning of rare species in ecological communities deserves special academic attention [1]. Our research demonstrates that a rare mammal, the black-and-white snub-nosed monkey, exerts important ecosystem functions in their native habitat. The monkeys have been shown to enhance forest structural heterogeneity and species diversity. We found that an adult monkey breaks 12 branches a day, so the breakage events are abundant considering the whole population and the time scale. These behaviors thus profoundly affect forest canopy structure, widen canopy gaps and thereby alter the climate conditions of the sub-canopy. In addition, they promote seed dispersal, resulting in a highly diverse plant community along both vertical and horizontal gradients and a pyramidal age structure, with a large cohort of young trees/saplings and few old trees. The role of *R. bieti* in seed dispersal through food intake is demonstrated by the composition of the motherless sapling pool in its current habitat matching well with its known diet. *R. bieti* mainly feed on *Acanthopanax gracilistylus, Sorbus thibetica, S. scalaris, Padus obtusata, Bothrocaryum controversum*, and *Meliosma yunnanensis*. These young plants were in the current habitat but were absent in the historical habitat. The role of *R. bieti* in increasing plant dispersion through expanding forest gaps is firstly reflected by the observation that, in the current habitat, there are more motherless saplings beyond the monkey’s diet, such as *Rhododendron basilicum, R. parvifolium, Quercus spinosa, Pinus armandi, Abies fabri, Tsuga chinensis, Acer pictum* and *Viburnum dilatatum*, while in the historical habitat, the abundance and species richness of motherless saplings were lower, only containing *Rhododendron basilicum, Abies fabri,* and *Tsuga chinensis*. Secondly, for the saplings with mother plants nearby, saplings were within a distance of 12 m from the mother plants (mean = 4.3 m, n = 101) in the current habitat, while in the historical habitat H-20, saplings were within a distance of 4 m from the mother plants (mean = 2.8 m, n = 121). The above changes induced by the monkeys’ activities led to a more heterogeneous vegetation structure, which can increase the resilience and robustness of this cold temperate coniferous forest ecosystem [28]. After the removal of this ‘ecosystem engineer’, these benefits can persist for a period of up to 20 years, as the density of trees and young saplings at H-10 and H-20 was not significantly lower than in the current habitat. Moreover, the monkeys utilize their habitat and food resources in a seemingly sustainable way by rotating the exploitation of areas of their home range [12,29].

Therefore, the monkeys contribute actively towards maintaining the sustainability of the forest ecosystem and should be given more attention by ecologists and conservationists. Seemingly, the red colobus of Kibale (*Procolobus rufomitratus*) can shift the composition of forests as engineers through their foraging behavior [30]. And numerous frugivorous primates can enhance forest regeneration through dispersing seeds [31]. If an ecosystem engineer disappears from an ecosystem, numerous other species will be affected. For example, Elephants (Elephantidae) are widely recognized as ecosystem engineers [32] and have been proven to have impacts at varying spatial scales [33]. Identifying and understanding the roles of ecosystem engineers is critical.

Using flagship or umbrella species as surrogates is an efficient shortcut for biodiversity conservation. However, there are several problems with its practical application, and the importance of surrogate species in biodiversity conservation is debated [34]. For example, the habitats of surrogate species (often rare ones) do not always adequately shelter sympatric species [35], resulting in the neglect of numerous species in need of protection [36], which is known as the “flagship species conundrum” [37]. Some scientists suggested dividing subspecies of flagship species into distinct species to raise public awareness [38], while others proposed “flagship fleets” that deploy multiple flagships in a single campaign [39]. However, these approaches would dilute the finite conservation resources and could be confusing to the public and less cost-effective [40]. The traditional concept of surrogate or umbrella species aims to maximize species coexistence [41], but it neglects the imbalance of ecological roles among species and does not incorporate the dynamics of ecosystems into conservation goals. In contrast, this study proposes a “function-oriented surrogate species” framework, which shifts the conservation goal from “species quantity priority” to “ecological function priority” by focusing on maintaining key ecological processes, thereby more directly achieving the ultimate biodiversity conservation goal of “self-sustaining ecosystems” [42].

A globally integrated map of flagship species should be developed to enhance conservation efficiency, with each geographic region’s ecosystem such as exemplified by the Global 200 framework [43] represented by a unique surrogate species. First, experts will nominate candidate flagship species based on regional ecological contexts to establish an initial list. Second, candidate species will undergo rigorous assessments of their ecological role to prioritize and finalize the list. Finally, a framework will be established for long-term population monitoring and ecosystem surveillance, implementing adaptive management strategies. This surrogate species should possess a specific ecological function that contributes to maintaining the health and sustainability of a particular ecosystem in that geographic region, such as the monkeys for the temperate coniferous forest ecosystem of the Yunling Mountains. When selecting a surrogate species, one should not only consider whether it is a keystone in the food web but also whether it has the ability to cause moderate disturbance to the ecosystem. Moderate disturbance is operationally defined as intermittent, non-lethal disturbances that maintain ecological heterogeneity without triggering ecosystem collapse [44]. This aligns with the intermediate disturbance hypothesis, which posits that diversity peaks at intermediate disturbance levels, balancing competitive exclusion and colonization opportunities [44]. Animals that provide appropriate disturbance can have a strong positive impact on ecosystem biodiversity and resilience, even if they are not keystone species in the food web. Studies have also revealed that biodiversity is generally higher in regions with moderate human disturbance [45,46]. Large-bodied, rare animals that provide appropriate disturbance to ecosystems could be prime candidates for surrogate species.

The points discussed above emphasize the need to intensify research on the ecological functions of species, especially large mammals. Although local extinctions are sad, they can also be an important opportunity to study the function of species, as in this study. In addition, even if an identified flagship species becomes locally extinct, this does not necessarily affect its role as a conservation surrogate in a particular ecosystem. Setting goals for ecosystem restoration has always been a challenge for conservation managers. The reintroduction and recovery of locally extinct flagship species should be prioritized as conservation surrogates to explicitly anchor ecosystem stability and resilience as restoration targets across both short-term and long-term timeframes. To ensure the ecological effectiveness of the reintroduction plan, it is necessary to prioritize the selection of donor populations that have the highest genetic similarity to the historical local population, and establish an ecological corridor network for gene exchange between populations. It is also necessary to confirm that the key habitats in the re-introduction area have been largely restored to the historical baseline level through existing conservation measures; to quantify the minimum viable population size of the target species and the threshold for their demand for key resources; and to ensure the supply of resources in the target area. In an age of rapid global environmental change, the selection and inventory of surrogate species for regional endemic ecosystems can also contribute to the achievement of global biodiversity conservation and sustainable development goals [47].

## 5. Conclusions

This study highlights the critical ecological role of the black-and-white snub-nosed monkey as an ecosystem engineer in temperate coniferous forests of the Yunling Mountains. Field observations and forest structural analysis prove that the monkey’s branch-breaking behavior have changed the forest structure obviously. These activities maintain a heterogeneous vegetation structure and a pyramidal age distribution of trees, thereby increasing ecosystem resilience and stability. Although the positive ecological effects of the monkeys can persist for up to 20 years after their disappearance, long-term absence results in reduced regeneration, sapling density, and overall forest health.

Beyond their local importance, our findings suggest that conservation strategies should move beyond traditional flagship or surrogate species concepts focused solely on species richness and instead emphasize species’ functional roles in sustaining ecosystems. In the future, large-bodied, rare animals that provide moderate disturbance, such as snub-nosed monkeys, should be prioritized as conservation surrogates because of their unique ability to maintain biodiversity and ecosystem resilience. Recovering locally extinct flagship species and incorporating them into ecosystem restoration plans could serve as a powerful conservation target, helping to achieve both regional sustainability and global biodiversity goals.

The ecological engineer effect of rare species revealed in this study has global significance that goes beyond the protection of individual species. With the intensification of climate change and the sharp decline in biodiversity, the traditional conservation model centered on species quantity or endangered status urgently needs to shift towards function-oriented ecosystem management. Incorporating the population recovery of “functional flagship species” into landscape-level ecological restoration plans can simultaneously enhance carbon sink capacity, maintain biodiversity, and improve the ecological well-being of communities.

## Data Availability

The original contributions presented in this study are included in the article/Supplementary Material. Further inquiries can be directed to the corresponding author(s).

## Supplementary Materials

The following supporting information can be downloaded at: https://www.mdpi.com/article/10.3390/ani15203021/s1, Figure S1: Study area showing the current habitat and historical habitats of the black-and-white snub-nosed monkey; Figure S2: The distribution of plant sampling quadrats (grey squares) along elevational belts in current and historical habitats; Figure S3: The height structure of trees in the current and historical habitats; Table S1: Current and historical locations of *Rhinopithecus bieti* populations and time since local extirpation; Table S2 Measurements of broken branches, canopy structure, under canopy conditions, and plant community structure in current and historical habitats; Table S3: The five most abundant trees, shrubs and herbs in current and historical habitats; Table S4: Statistical details for the comparison of plant community structure, length and diameter of broken branches, and biomass of *Usnea longissima* between current and historical habitats.

## Abbreviations

The following abbreviations are used in this manuscript:

IUCN	International Union for Conservation of Nature
H-0	Current habitat
H-10	Habitat where local extinction occurred around 10 years ago
H-20	Habitat where local extinction occurred around 20 years ago
H-30	Habitat where local extinction occurred around 30 years ago
H-40	Habitat where local extinction occurred around 40 years ago
