# Supplementary material for: Rare Primate Rhinopithecus bieti Can Sustain the Resilience of Montane Forests
Source: Animals (Basel). 2025 Oct 17;15(20):3021. doi: 10.3390/ani15203021 (PMC12561596; doi:10.3390/ani15203021)
Supplement: Supplementary file 1 [file animals-15-03021-s001.zip › animals-3876246-supplementary.pdf]

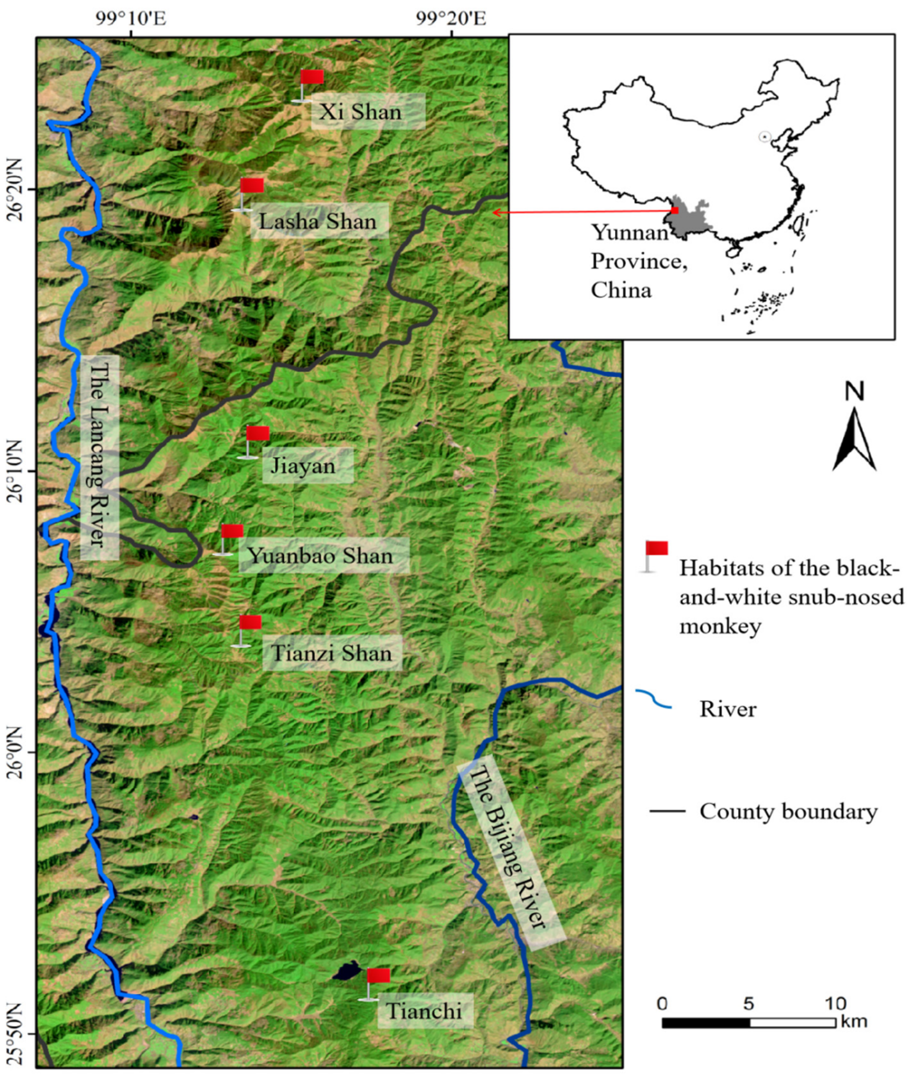

Figure S1 Study area showing the current habitat and historical habitats of the black-and-white snub-nosed monkey. Lasha Shan constitutes the current habitat (H-0). The population at Jiayan (H-10) vanished approximately 10 years before 2019, and those at Yuanbao Shan (H-20), Tianzi Shan (H-30), Tianchi (H-40) and Xi Shan (H-40) became extirpated approximately 20, 30, 40 and 40 years ago, respectively.

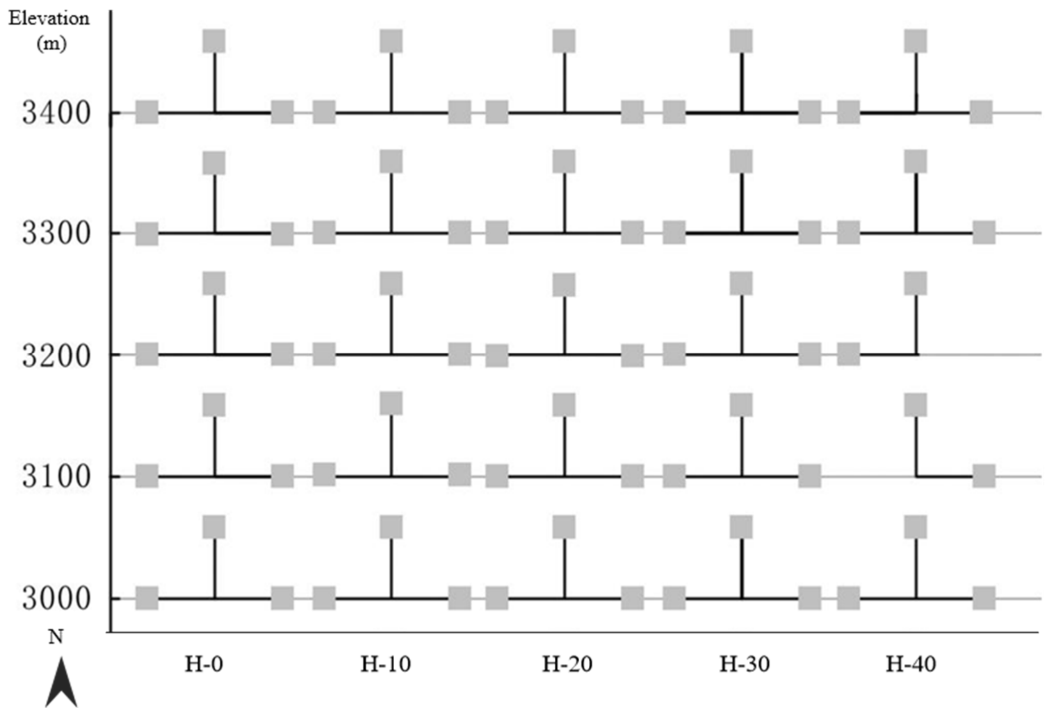

Figure S2 The distribution of plant sampling quadrats (grey squares) along elevational belts in current and historical habitats. In each quadrat, we measured biomass of *Usnea longissima,* broken branches, canopy structure (the size of canopy gaps and canopy diameter), and plant community structure (species diversity, abundance, and height of trees, shrubs and herbs; diameter at breast height of trees; age of trees; number of hollow trees). Two quadrats were missing in H-40, one on the western slope at 3100 m and one on the eastern slope at 3200 m.

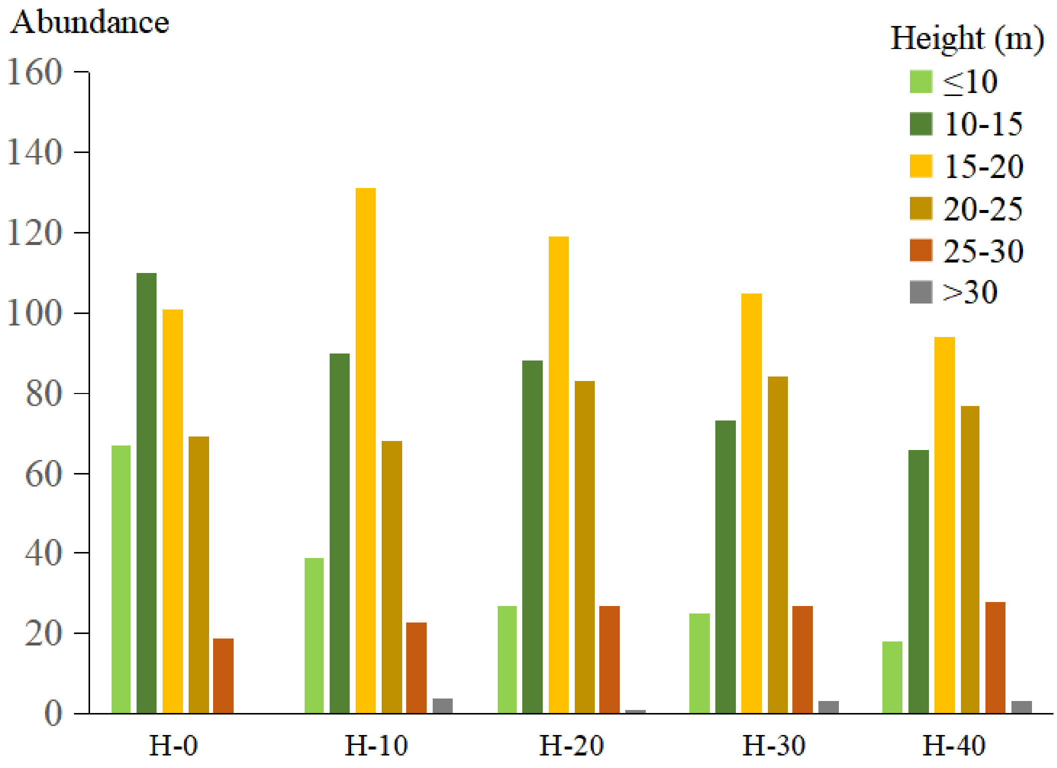

Figure S3 The height structure of trees in the current and historical habitats. The height of trees was divided into 6 categories: ≤10 m, 10-15 m, 15-20 m, 20-25 m, 25-30 m and >30 m.

Table S1 Current and historical locations of *Rhinopithecus bieti* populations and time since local extirpation.

Location/population	Abbreviation	Population stabilization period and population size	Year monkey presence was recorded for the last time
Lasha Shan	H-0	Present, ~100 individuals	Present
Jiayan	H-10	Before 2008, ~100 individuals	2008
Yuanbao Shan	H-20	1970s, ~30 individuals	1997
Tianzi Shan	H-30	1970s, ~30 individuals	1989
Tianchi	H-40	1960s, ~30 individuals	1980
Xi Shan		1960s, ~30 individuals	

Table S2 Measurements of broken branches, canopy structure, under canopy conditions, and plant community structure in current and historical habitats.

Items	Sub items	H-0	H-10	H-20	H-30	H-40
Broken branches	Number	55	62	64	72	76
	Average diameter (cm)	3.59	3.21	2.48	1.98	1.79
	Average length (cm)	109.96	98.98	75.19	61.26	49.36
Canopy structures	No. of measured trees (DBH>10cm)	366	355	345	317	286
	Average canopy diameter (m)	3.39	4.01	4.54	4.98	5.15
	Average canopy gap (%)	38.30	33.48	32.85	31.39	29.87
Environment condition of under canopy	Solar radiation (KLux)	3.03	2.00	1.70	1.27	1.40
	Air temperature (°F)	50.64	51.72	51.11	50.89	50.42
	Air moisture (%)	85.90	87.49	87.12	86.73	84.64
Vertical structure of forests	Averaged height of trees (m)	15.65	16.80	17.48	17.93	18.27
	Averaged height of shrubs (m)	1.61	1.44	1.32	1.23	1.26
	Averaged height of herbs (cm)	16.50	14.23	12.40	11.48	10.03
Plant species diversities	Species richness of trees	46	27	20	16	24
	Species richness of shrubs	61	44	30	18	26
	Species richness of herbs	40	23	21	16	14
	Abundance of trees	366	355	345	317	286
	Abundance of shrubs (and coverage)	851 (12.99%)	620 (10.25%)	429 (7.79%)	384 (6.94%)	379 (6.75%)
	Abundance of herbs (and coverage)	1068 (24.42%)	565 (21.35%)	550 (21.85%)	501 (26.89%)	503 (26.69%)
Demographic structure of trees	Averaged DBH of trees (cm)	29.84	34.46	36.49	37.95	38.80
	Abundance of hollow trees	3	16	17	23	29
	Abundance of motherless saplings (and proportion to all saplings)	47 (37.01%)	10 (9.62%)	3 (3.06%)	2 (2.30%)	4 (5.19%)

Table S3 The five most abundant trees, shrubs and herbs in current and historical habitats. Abundance refers to the number of stems.

Habitats	Trees		Shrubs		Herbs	
	Species name	Abundance	Species name	Abundance	Species name	Abundance
H-0	*Abies georgei*	61	*Fargesia dura*	53	*Sphagnum palustre*	55
	Acanthopanax evodiaefolius	45	*Fargesia spathacea*	44	*Duchesnea indica*	50
	Tsuga dumosa	37	*Rhododendron faberi*	11	*Pteris cretica var.nervosa*	27
	Rhododendron mucronatum	23	*Nothopanax delavayi*	9	*Liparis nervosa*	16
	Symplocos heishanensis	21	*Berberis mitifolia*	8	*Ainsliaea foliosa*	12
H-10	*Tsuga dumosa*	91	*Fargesia papyrifera*	36	*Sphagnum palustre*	61
	Larix gmelinii	61	*Fargesia spathacea*	32	*Duchesnea indica*	37
	Rhododendron faberi	34	*Rhododendron faberi*	26	*Pteris cretica var. nervosa*	26
	Rhododendron oreodoxa	34	*Lonicera buchananii*	9	*Ainsliaea foliosa*	15
	Lithocarpus variolosus	27	*Berberis mitifolia*	9	*Micranthemum micranthemoides*	12
H-20	*Tsuga dumosa*	78	*Rhododendron faberi*	36	*Sphagnum palustre*	65
	Abies georgei	51	*Fargesia spathacea*	33	*Duchesnea indica*	50
	Lithocarpus variolosus	40	*Fargesia papyrifera*	33	*Pteris cretica var. nervosa*	23
	Acanthopanax evodiaefolius	35	*Berberis mitifolia*	18	*Duchesnea indica*	11
	Rhododendron faberi	24	*Cotoneaster acuminatus*	14	*Ainsliaea foliosa*	11
H-30	*Tsuga dumosa*	86	*Rhododendron faberi*	45	*Sphagnum palustre*	58
	Abies georgei	68	*Fargesia papyrifera*	36	*Duchesnea indica*	48
	Rhododendron faberi	41	*Fargesia spathacea*	19	*Pteris cretica var. nervosa*	26
	Acanthopanax evodiaefolius	29	*Berberis mitifolia*	18	*Ainsliaea foliosa*	13
	Lithocarpus variolosus	23	*Cotoneaster acuminatus*	16	*Agrimonia pilosa*	12

H-40	*Abies georgei*	60	*Fargesia spathacea*	37	*Sphagnum palustre*	65
	Tsuga dumosa	43	*Rhododendron faberi*	27	*Duchesnea indica*	50
	Rhododendron faberi	38	*Fargesia dura*	22	*Pteris cretica var.nervosa*	15
	Acanthopanax evodiaefolius	28	*Berberis mitifolia*	18	*Ainsliaea foliosa*	13
	Rhododendron mucronatum	21	*Cotoneaster horizontalis*	14	*Rubus parvifolius*	11

Table S4 Statistical details for the comparison of plant community structure, length and diameter of broken branches, and biomass of *Usnea longissima* between current and historical habitats.

Content	Object (Statistics method)	Habitat	H-0
Difference of broken branches caused by the monkey and other factor	Length of broken branches (Welch two sample t-test)	H-10	t = 1.7861, df = 84.127, P = 0.03885*
		H-20	t = 4.763, df = 67.681, P = 5.233e-06****
		H-30	t = 6.8485, df = 61.518, P = 2.033e-09****
		H-40	t = 8.7274, df = 56.147, P = 2.442e-12****
	Diameter of broken branches (Welch two sample t-test)	H-10	t = 1.4799, df = 111.05, P = 0.07086·
		H-20	t = 5.3427, df = 76.809, P = 4.53e-07****
		H-30	t = 8.1362, df = 65.409, P = 8.018e-12****
		H-40	t = 9.3501, df = 58.514, P = 1.62e-13****
Figure 2 (A)	Crown diameters (Welch two sample t-test)	H-10	t = -7.6234, df = 718.98, P = 3.915e-14****
		H-20	t = -14.729, df = 706.81, P < 2.2e-16****
		H-30	t = -19.584, df = 677.53, P < 2.2e-16****
		H-40	t = -18.663, df = 567, P < 2.2e-16****
Figure 2 (B)	Canopy gaps (Welch two sample t-test)	H-10	t = 2.4535, df = 75.294, P = 0.008231**
		H-20	t = 3.1099, df = 66.441, P = 0.001378**
		H-30	t = 4.4092, df = 49.979, P = 2.757e-05****
		H-40	t = 4.2044, df = 72.848, P = 3.672e-05****
Figure 2 (C)	Solar radiation (Welch two sample t-test)	H-10	t = 15.45, df =34, P < 0.0001****
		H-20	t = 19.15, df =34 , P < 0.0001****
		H-30	t =26.19 , df = 34, P < 0.0001****
		H-40	t = 23.43, df =34, P < 0.0001****
Figure 2 (D)	Height of trees (Welch two sample t-test)	H-10	t = -2.7985, df = 718.27, P = 0.002636**
		H-20	t = -4.5608, df = 709, P = 3.001e-06****
		H-30	t = -5.4603, df = 671.37, P = 3.348e-08****
		H-40	t = -6.1137, df = 620.54, P = 8.61e-10****
	Height of shrubs (Welch two sample t-test)	H-10	t = 4.2409, df = 487.49, P = 1.332e-05****
		H-20	t = 6.9499, df = 428.02, P = 6.851e-12****
		H-30	t = 9.2697, df = 413.67, P < 2.2e-16****
		H-40	t = 8.645, df = 420.53, P < 2.2e-16****
	Height of Grass (Welch two sample t-test)	H-10	t = 2.486, df = 496.99, P = 0.006623**
		H-20	t = 4.5377, df = 489.99, P = 3.581e-06****
		H-30	t = 5.6966, df = 472.83, P = 1.075e-08****
		H-40	t = 7.6054, df = 465.44, P = 7.924e-14****
Figure 2 (E)	Species richness of trees (Paired Wilcoxon signed rank test)	H-10	V = 66, P = 0.001825**
		H-20	V = 78, P = 0.001178**
		H-30	V = 91, P = 0.0007932***
		H-40	V = 78, P = 0.00122**
	Species richness of shrubs (Paired Wilcoxon signed	H-10	V = 79, P = 0.01012*
		H-20	V = 66, P = 0.00175**

	rank test)	H-30	V = 91, P = 0.0008146***
		H-40	V = 78, P = 0.001234**
	Species richness of grass (Paired Wilcoxon signed rank test)	H-10	V = 91, P = 0.0008119***
		H-20	V = 91, P = 0.0008119***
		H-30	V = 91, P = 0.0007985***
		H-40	V = 91, P = 0.0007906***
Figure 2 (F)	Density of trees (Paired Wilcoxon signed rank test)	H-10	V = 39, P = 0.3115
		H-20	V = 32, P = 0.1425
		H-30	V = 85, P = 0.002972**
		H-40	V = 91, P = 0.0008065***
	Density of shrubs (Paired Wilcoxon signed rank test)	H-10	V = 73, P = 0.004231**
		H-20	V = 89, P = 0.001312**
		H-30	V = 91, P = 0.0008146***
		H-40	V = 91, P = 0.0008146***
	Density of grass (Paired Wilcoxon signed rank test)	H-10	V = 91, P = 0.000788***
		H-20	V = 89.5, P = 0.001147**
		H-30	V = 91, P = 0.0008065***
		H-40	V = 91, P = 0.0008146***
Figure 4 (A)	Diameter at breast height of trees (Wilcoxon signed rank test)	H-10	W = 84207, P = 1
		H-20	W = 39290, P < 2.2e-16****
		H-30	W = 31624, P < 2.2e-16****
		H-40	W = 26242, P < 2.2e-16****
Figure 4 (D)	Proportion of hollow trees (Paired Wilcoxon signed rank test)	H-10	V = 2, P = 0.005413**
		H-20	V = 0, P = 0.004576**
		H-30	V = 0, P = 0.002945**
		H-40	V = 1, P = 0.002528**
Figure 4 (E)	Density of young saplings (Paired Wilcoxon signed rank test)	H-10	V = 51.5, P = 0.0537·
		H-20	V = 78, P = 0.01231*
		H-30	V = 73, P = 0.004106**
		H-40	V = 75.5, P = 0.002268**
Figure 4 (F)	Proportion of motherless young saplings (Paired Wilcoxon signed rank test)	H-10	V = 83, P = 0.003052**
		H-20	V = 66, P = 0.00192**
		H-30	V = 66, P = 0.00192**
		H-40	V = 63, P = 0.004343**

Notes: ****: P<0.0001 ***: P<0.001 **: P<0.01 *: P<0.05 · : P<0.1
